# Caffeine at a Moderate Dose Did Not Affect the Skeletal System of Rats with Streptozotocin-Induced Diabetes

**DOI:** 10.3390/nu9111196

**Published:** 2017-10-30

**Authors:** Joanna Folwarczna, Aleksandra Janas, Urszula Cegieła, Maria Pytlik, Leszek Śliwiński, Magdalena Matejczyk, Anna Nowacka, Karolina Rudy, Zora Krivošíková, Kornélia Štefíková, Martin Gajdoš

**Affiliations:** 1 Department of Pharmacology, School of Pharmacy with the Division of Laboratory Medicine in Sosnowiec, Medical University of Silesia, 40-055 Katowice, Poland; ajanas@sum.edu.pl (A.J.); ucegiela@o2.pl (U.C.); mariapytlik@gmail.com (M.P.); lsliw@o2.pl (L.Ś.); magdamatejczyk@poczta.onet.pl (M.M.); anulkanow4@wp.pl (A.N.); rudy.karolina@gmail.com (K.R.); 2 Department of Clinical and Experimental Pharmacotherapy, Medical Faculty, Slovak Medical University, 833 03 Bratislava, Slovakia; zora.krivosikova@szu.sk (Z.K.); kornelia.stefikova@szu.sk (K.Š.); martin.gajdos@szu.sk (M.G.)

**Keywords:** caffeine, streptozotocin, diabetes, skeletal system, rats

## Abstract

Diabetes may lead to the development of osteoporosis. Coffee drinking, apart from its health benefits, is taken into consideration as an osteoporosis risk factor. Data from human and animal studies on coffee and caffeine bone effects are inconsistent. The aim of the study was to investigate effects of caffeine at a moderate dose on the skeletal system of rats in two models of experimental diabetes induced by streptozotocin. Effects of caffeine administered orally (20 mg/kg aily for four weeks) were investigated in three-month-old female Wistar rats, which, two weeks before the start of caffeine administration, received streptozotocin (60 mg/kg, intraperitoneally) alone or streptozotocin after nicotinamide (230 mg/kg, intraperitoneally). Bone turnover markers, mass, mineral density, histomorphometric parameters, and mechanical properties were examined. Streptozotocin induced diabetes, with profound changes in the skeletal system due to increased bone resorption and decreased bone formation. Although streptozotocin administered after nicotinamide induced slight increases in glucose levels at the beginning of the experiment only, slight, but significant unfavorable changes in the skeletal system were demonstrated. Administration of caffeine did not affect the investigated skeletal parameters of rats with streptozotocin-induced disorders. In conclusion, caffeine at a moderate dose did not exert a damaging effect on the skeletal system of diabetic rats.

## 1. Introduction

Dietary factors and lifestyle may significantly affect the development of chronic diseases, including type 2 diabetes and osteoporosis. In type 2 diabetes, progressive defects of insulin secretion occur, usually against the background of insulin resistance, whereas type 1 (insulin-dependent) diabetes is characterized by autoimmune β-cell destruction and insulin deficiency [1]. Both diabetes types may be associated with development of disorders of bone metabolism and increased fracture risk [2,3].

The mechanism of development of osteoporotic changes in diabetes is very complex. There are three types of bone cells (osteoblasts, osteocytes and osteoclasts); all of them are affected by hyperglycemia and the inflammatory environment [4]. The unfavorable effects of diabetes on bone include, among others, attenuation of stimulatory effect of insulin on osteoblasts due to insulin deficiency, increased expression of sclerostin in osteocytes, intensification of osteoclast activity, and decreased quality of bone due to production of advanced glycation products [4,5]. Increased oxidative stress, occurring in both types of diabetes [2,6], also may lead to intensification of bone resorption and inhibition of bone formation [7]. In type 1 diabetes, bone mineral density (BMD) decreases and the risk of fracture increases. In type 2 diabetes, the fracture risk is also increased, but a reduction in BMD does not occur [2,3,5,8,9,10].

The well-established experimental model of experimental type 1 diabetes is diabetes induced in rats and mice by a single injection of streptozotocin, selectively accumulating in pancreatic β-cells and destroying them [11,12]. Contrary to type 1 diabetes, the available rat models of type 2 diabetes are not fully adequate [13]. One such model is a model of diabetes induced by streptozotocin administered to the rats after nicotinamide, which protects β cells, weakening the effects of streptozotocin; according to the literature the rats demonstrate features of type 2 diabetes [14].

Epidemiological studies suggest that coffee consumption may reduce the incidence of type 2 diabetes [15,16]. The coffee effects on the skeletal system are not clear; there are reports indicating an increased risk of bone fractures [17], no effect [17,18,19], and even some beneficial bone effects [20,21,22,23]. There are no clinical data on the effect of coffee consumption on the development of diabetes-induced skeletal disorders. The most known active constituent of coffee is caffeine. Caffeine consumption varies throughout the world. Its main dietary source is usually coffee, but in Great Britain and Japan the intake is mainly through tea, and in Argentina through yerba mate [15,24]. Caffeine is also present in caffeinated soft drinks. It is difficult to estimate the caffeine intake, because there are significant differences in caffeine content in different products [25]. Nevertheless, caffeine is present in the human diet on a daily basis, including for patients with type 1 or type 2 diabetes. Although acute caffeine administration leads to reduced insulin sensitivity [15,26], caffeine may contribute to the effects of regular coffee use associated with decreasing the incidence of type 2 diabetes [16].

Data on the effect of caffeine on bone in experimental conditions are inconsistent. Although the majority of previously published reports on the studies of caffeine administered at different doses for various periods of time to rats demonstrated rather its adverse skeletal effects [27,28,29,30,31,32,33,34,35,36,37,38,39,40,41,42], we have shown that caffeine administration at a moderate dose of 20 mg/kg per os (p.o.) once daily (by oral gavage) slightly counteracted the development of osteoporosis induced by estrogen deficiency [25]. The inconsistent skeletal effects reported for caffeine might result from differential caffeine effects in different animal models. The effects of caffeine on the skeletal system of rats with experimental diabetes have never been investigated, although the results might be relevant to the caffeine effects in diabetic patients.

The research hypothesis of the present study was that caffeine may affect the rat skeletal system in diabetic conditions. The aim of the study was to investigate effects of caffeine at a moderate dose on the skeletal system of rats with experimental type 1 and type 2 diabetes. We evaluated the effects of caffeine at the same dose as in our previous study [25]. It was estimated, taking into account species differences in caffeine disposition, that a caffeine dose of 20 mg/kg in the diet corresponds to a human consumption of approximately two cups of coffee daily [31]. The effects of caffeine were studied in two experimental models: rats treated with a single dose of streptozotocin, which induced experimental type 1 diabetes [12], and rats treated with nicotinamide and then streptozotocin [14], in which a slight, short-term increase in mean blood glucose level only was induced. Although the rats did not develop type 2 diabetes, some unfavorable skeletal effects were demonstrated. We decided to present here the results concerning the effects of caffeine in the nicotinamide/streptozotocin-treated rats, because the model allowed us to investigate the caffeine effects in rats with slight bone disorders, which might have been triggered by a short-term hyperglycemia. Since only the effects of one caffeine dose were examined, the study must be considered as preliminary.

## 2. Materials and Methods

### 2.1. Animals and Chemicals

The experiments were carried out on three-month-old (young mature) female Wistar rats obtained from the Center of Experimental Medicine, Medical University of Silesia, Katowice, Poland. The rats were fed a standard laboratory diet (Labofeed B, Wytwórnia Pasz “Morawski”, Kcynia, Poland) ad libitum. The animals were kept under monitored standard laboratory conditions complying to the European Union guidelines (directive 2010//63/EU), in standard plastic cages (Tecniplast, Buguggiate, Italy), under a 12-h light–12-h dark cycle. The experimental protocol was approved by the Local Ethics Commission, Katowice, Poland (permission number 81/2013).

The drugs used included: caffeine (Sigma-Aldrich Co., St. Louis, MO, USA) at a dose of 20 mg/kg p.o. daily for four weeks, streptozotocin (Cayman Chemical Company, Ann Arbor, MI, USA), nicotinamide (Sigma-Aldrich Co., St. Louis, MO, USA), ketamine (Bioketan; Vetoquinol Biowet Sp. z o.o., Gorzów Wielkopolski, Poland), xylazine (commercial name Xylapan; Vetoquinol Biowet Sp. z o.o., Gorzów Wielkopolski, Poland).

At the start of the study, the animals were divided into five groups (*n* = 10): control rats (I), streptozotocin-treated control rats (II), streptozotocin-treated rats receiving caffeine (20 mg/kg p.o. daily) (III), nicotinamide/streptozotocin-treated control rats (IV), and nicotinamide/streptozotocin-treated rats receiving caffeine (20 mg/kg p.o. daily) (V).

Streptozotocin was administered to the rats of groups II–V once, at a dose of 60 mg/kg intraperitoneally (i.p.), dissolved in 0.1 M citrate buffer (pH 4.5; 1 mL/kg) [12]. Nicotinamide, at a dose of 230 mg/kg i.p., dissolved in 0.9% NaCl (2 mL/kg), was injected 15 min before the administration of streptozotocin in the rats of groups IV and V [14]. Control rats of group I received the citrate buffer in a volume of 1 mL/kg i.p. Administration of caffeine, once daily by oral gavage, started two weeks after the streptozotocin injection and lasted four weeks. The control rats (groups I, II and IV) received the vehicle (tap water) at the same volume of 2 mL/kg p.o. This study shared controls with our previous report [43]. The four-week period of caffeine administration was long enough to demonstrate effects of caffeine and other plant compounds on the skeletal system in rats [25,44,45]. It should be pointed out that four weeks in the life of the rat correspond to more than two years in the human life span [46].

The blood samples were taken from tail vessels of conscious rats (by cutting the tail tip) at the start of the experiment and then once weekly, before caffeine administration, in order to examine the non-fasting blood glucose with the use of Accu-Chek Performa glucometer (Roche Diagnostics GmbH, Mannheim, Germany). When the glucose level exceeded 600 mg/100 mL (the upper limit of detection), this value was taken into the calculations. The streptozotocin-treated rats of groups II and III, which did not develop diabetes were excluded from the experiment. One rat died during the experiment. The final number of rats per group was: 10 in groups I, IV, and V, and 8 in groups II and III.

The rats were fasted overnight after the last caffeine or vehicle administration. The next day, the rats were anesthetized (ketamine and xylazine), and sacrificed by cardiac exsanguination. The left and right femurs, left tibia, and L-4 vertebra were isolated. The left femurs and tibias, and vertebrae, were weighed. The left femur, left tibia, and proximal part of the right femur from each rat, wrapped in gauze soaked in 0.9% NaCl solution, were kept below −20 °C until the mechanical tests were carried out on thawed bones [47].

### 2.2. Bone Mechanical Property Studies

Bone mechanical properties were determined with the use of an Instron 3342 500N apparatus (Instron, Norwood, MA, USA). The data were analyzed by Bluehill 2 version 2.14 software (Instron, Norwood, MA, USA).

Mechanical properties of the proximal metaphysis of the left tibia were measured in a three-point bending test as previously described [25,48]. The proximal epiphysis of the tibia was removed before the test. The load, displacement and energy for the yield point (0.05% offset), maximum load point and fracture load point were determined based on the load-displacement curves obtained for each bone. The intrinsic parameters (stress and Young’s modulus) were examined, assuming that the tibial metaphysis was a circular beam [25].

Mechanical properties of the diaphysis of the left femurs were determined using a three-point bending test as previously described [25,47]. The load was applied perpendicularly to the long axis of the femur at the mid-length of the bone. The same parameters as for the tibial metaphysis were determined. To calculate the intrinsic parameters, it was assumed that the femoral diaphysis was an elliptical pipe, as previously described [25].

Mechanical properties of the femoral neck were assayed in a compression test [25]. The load was applied to the femoral head along the long axis of the bone. The load causing the fracture of the femoral neck (maximum load) was determined.

### 2.3. Bone Mineralization Studies

The left tibias, femurs, and L-4 vertebra were lyophilized using FreeZone 6 lyophilizer (−51 °C, 0.03 mBa; Labconco, Kansas City, MO, USA), for nine days and weighed. Bone mineral density (BMD), bone mineral content (BMC), and bone area measurements were carried out on the lyophilized tibias deprived of the proximal epiphysis and on the L-4 vertebra, using dual energy X-ray absorptiometry (DXA; Lunar Prodigy Advance with Encore 2011 software version 13.60, GE Medical Systems, Madison, WI, USA). All samples were measured for three times and the average value was used for further calculations.

The lyophilized bones were then mineralized at 640 °C for 48 h in a muffle furnace L9/11/C6 (Nabertherm, Lilienthal, Germany). The content of bone mineral, bone organic substances, and bone water in the bones were calculated as the ratios to the bone mass.

### 2.4. Histomorphometric Studies

The histomorphometric measurements were performed on undecalcified slides prepared from the right tibias and femurs, using Lucia G version 4.51 software (Laboratory Imaging, Praha, Czech Republic) and OsteoMeasure XP v1.3.0.1 software (OsteoMetrics, Inc., Decatur, GA, USA), as previously described [25,45,49].

The area of the transverse cross-section of the cortical bone and the area of the transverse cross-section of the marrow cavity of the femoral diaphysis (in the bone midlength) and the tibial diaphysis (close to the point where the fibula grows into it) were determined. The width of epiphyseal and metaphyseal trabeculae, as well as the width of the epiphyseal cartilage were determined in longitudinal preparations of the distal femoral epiphysis.

### 2.5. Biochemical Studies

In the serum obtained on rat sacrifice, concentrations of osteocalcin (a bone formation marker) and C-terminal type I collagen fragments released from bone during bone resorption were determined using enzyme immunoassays (Rat-MID Osteocalcin EIA and RatLaps EIA, respectively, Immunodiagnostic Systems Ltd., Boldon, Tyne and Wear, UK), following the manufacturer’s instructions. Serum levels of fructosamine, total calcium, and total cholesterol were measured spectrophotometrically, using Pointe Scientific, Inc., Canton, MI, USA, kits, according to the manufacturer’s instructions.

### 2.6. Statistical Analysis

Results are presented as the mean ± standard error of the mean (SEM). Statistical analysis was carried out with the use of Kruskal–Wallis ANOVA followed by Mann–Whitney U test, because not all data met the ANOVA assumptions of homogeneity of variance (Levene’s test) or normality (Shapiro–Wilk’s test) (Statistica 12; StatSoft Polska Sp. o.o., Kraków, Poland). The results obtained in caffeine-treated rats (group III and V) were compared with those of the appropriate control rats treated with streptozotocin (groups II and IV, respectively). Results from all groups were also compared with those of the healthy control rats (group I). *p* values < 0.05 were considered significant.

## 3. Results

### 3.1. Effect of Caffeine on the Skeletal System of Rats with Streptozotocin-Induced Diabetes

**Figure 1:**
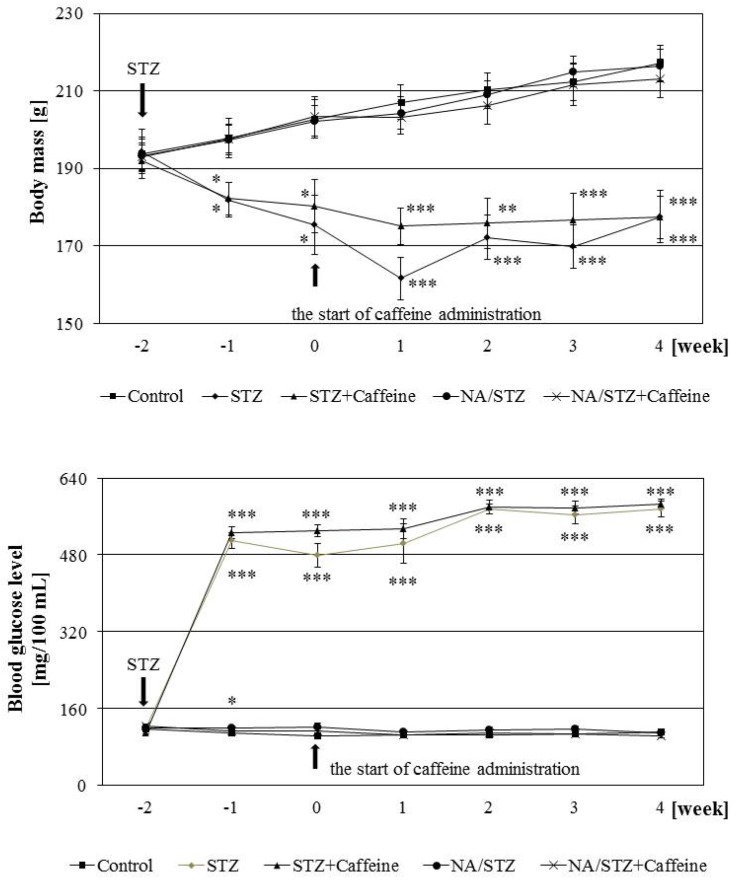
Effect of caffeine administered orally (20 mg/kg daily for four weeks) on the body mass and non-fasting blood glucose concentration in rats treated with streptozotocin (STZ) or nicotinamide and streptozotocin (NA/STZ). Results are presented as means ± SEM (standard error of the mean; *n* = 8–10). The final measurements of the body mass and blood glucose concentration were made before the last caffeine/vehicle administration. Kruskal–Wallis ANOVA followed by Mann–Whitney U test were used for evaluation of the significance of the results. * *p* < 0.05, ** *p* < 0.01, *** *p* < 0.001—in comparison with the control group.

**Figure 2:**
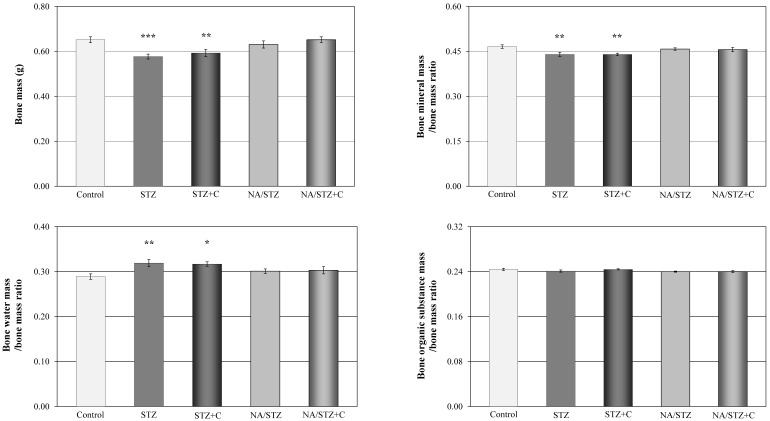
Effect of caffeine administered orally (C; 20 mg/kg daily for four weeks) on mass and composition of the femur in rats treated with streptozotocin (STZ) or nicotinamide and streptozotocin (NA/STZ). Results are presented as means ± SEM (standard error of the mean; *n* = 8–10). Kruskal–Wallis ANOVA followed by Mann–Whitney U test were used for evaluation of the significance of the results. * *p* < 0.05, ** *p* < 0.01, *** *p* < 0.001—in comparison with the control group.

Streptozotocin administration induced diabetes, with the non-fasting blood glucose concentrations higher than 400 mg/100 mL (*p* < 0.001, Figure 1). The serum level of fructosamine after six weeks was significantly increased in relation to the control rats (Table 1). The body mass of the diabetic rats was lower than in the control healthy rats (Figure 1), and the following osteoporotic changes developed in the skeletal system, as previously reported [43]: decreased bone mass and mineralization (Figure 2, Table 2), as well as decreased bone strength (Table 3). The serum biochemical marker of bone formation (osteocalcin) was decreased and the marker of bone resorption (RatLaps) increased (Table 1) in comparison with the healthy controls. Histomorphometric measurements in the control streptozotocin-treated rats indicated a tendency towards decreased bone formation (insignificant decrease in the transverse cross-sectional area of the cortical bone) and increased bone resorption (significant increase in the ratio of the transverse cross-sectional marrow cavity area to the diaphysis area) in the tibial diaphysis. Similar results were obtained in the femoral diaphysis (not shown). The width of epiphyseal cartilage significantly decreased in the diabetic rats. No significant changes were demonstrated in the width of epiphyseal and metaphyseal trabeculae in the femur.

Administration of caffeine (20 mg/kg p.o. for four weeks) did not affect glucose and fructosamine levels, as well as the body mass gain, in the diabetic rats (Table 1, Figure 1). There were no caffeine effects on the serum levels of total cholesterol and calcium (not affected by diabetes). There were no significant effects of caffeine on the levels of serum bone turnover markers in relation to the control diabetic rats. After caffeine administration, the level of the marker of bone resorption remained significantly increased in relation to that of the healthy control rats. The level of osteocalcin (bone formation marker) after administration of caffeine was not significantly different from both the diabetic and healthy controls.

Administration of caffeine did not affect femoral bone mass (Figure 2), and BMC and BMD of the tibia and vertebra (Table 2) in streptozotocin-induced diabetic rats, which remained significantly decreased in relation to the healthy control rats. There was also no effect of caffeine on the bone mineral mass to bone mass ratio (decreased in diabetes) and the bone water mass to bone mass ratio (increased in diabetes) in the bones (Figure 2).

Caffeine administration did not significantly affect mechanical properties of the proximal tibial metaphysis (mostly cancellous bone) in relation to the control diabetic rats, which strongly deteriorated in comparison with the healthy controls (Table 3). There was also no effect of caffeine on the diabetes-induced changes in mechanical properties of the femoral diaphysis (compact bone), which were much weaker than those observed in the tibial metaphysis, and involved decreases in the yield point load and energy accumulated to the yield point only (not shown). There were no changes observed in the strength of the femoral neck.

Caffeine administration did not significantly affect the histomorphometric parameters of both compact and cancellous bone of diabetic rats (Table 4).

### 3.2. Effect of Caffeine on the Skeletal System of Rats Treated with Nicotinamide and Streptozotocin

Administration of nicotinamide (230 mg/kg i.p.) 15 min before the injection of streptozotocin counteracted the development of diabetes. As previously reported [43], control nicotinamide/streptozotocin-treated rats had only increased mean blood concentration in the first days of the experiment in relation to the healthy controls (*p* < 0.05 one week after streptozotocin administration, Figure 1). There was no effect on the fructosamine level and body mass gain (Figure 1, Table 1). After six weeks from nicotinamide and streptozotocin administration, some slight, but significant changes in the skeletal system of the control nicotinamide/streptozotocin-treated rats were demonstrated, as follows: decreased vertebral BMD (Table 2), increased ratio of the bone water mass to bone mass in the tibia (not shown), and worsening of bone mechanical properties (Table 3).

Caffeine administration (20 mg/kg p.o. for four weeks) for rats treated with nicotinamide and streptozotocin did not affect the skeletal parameters. There was no caffeine effect on those skeletal parameters, which were significantly affected by nicotinamide and streptozotocin administration: the vertebral BMD (Table 2), the ratio of the bone water mass to bone mass in the tibia (not shown), and mechanical properties of the proximal tibial metaphysis (a decrease in the maximum load; Table 3) and femoral diaphysis (decreases in the yield point load and energy; not shown). There were also no statistically significant effects on bone histomorphometric parameters, neither of nicotinamide and streptozotocin administration in relation to the healthy controls, nor of caffeine administration in relation to the nicotinamide and streptozotocin-treated rats (Table 4).

## 4. Discussion

Although moderate coffee drinking is generally considered to be health-promoting [17,50], its effect on the skeletal system remains a matter of debate. One may speculate that the favorable effects of coffee/caffeine on the development of diabetes may be outweighed by the detrimental effect on bones. To investigate the effects of caffeine on bones in experimental diabetes, two rat models were used.

The model of type 1 diabetes induced by a single administration of streptozotocin in rats is one of the most widely used experimental diabetes models. Streptozotocin causes destruction of pancreatic β cells and severe hyperglycemia [12]. Accordingly, in the present study, the streptozotocin-treated rats developed diabetes, with profound changes in the skeletal system, in accordance with previous studies [51,52,53]. As previously reported [43], bone mineralization and mechanical properties of cancellous bone of the proximal tibial metaphysis strongly deteriorated. Bone resorption was intensified, and bone formation was decreased, as indicated by measurements of serum bone turnover marker concentrations and of the histomorphometric parameters of the cortical bone in the tibial diaphysis.

The model of nicotinamide/streptozotocin-treated rats has previously been reported to demonstrate some features corresponding to type 2 diabetes [13,54]. Nicotinamide administered 15 min before streptozotocin dose-dependently protects against the streptozotocin-induced destruction of β cells [14]. However, in the present study, nicotinamide at a dose of 230 mg/kg almost completely prevented the streptozotocin effect, and only slight increases in the mean blood glucose concentration were observed at the beginning of the experiment [43]. Also, fructosamine levels measured at the end of the experiment were not affected. Nevertheless, although diabetes did not develop, slight but significant decrease in vertebral BMD and worsening of mechanical properties of both cancellous and compact bone occurred in the nicotinamide/streptozotocin-treated control rats. The slight skeletal effects observed after nicotinamide/streptozotocin administration might have been a result of disorders of glucose metabolism, but the direct damaging effect of streptozotocin on rat bones cannot be excluded [43].

In the present study, no damaging effect of caffeine administered at a moderate dose of 20 mg/kg p.o. for four weeks on the skeletal system of rats with metabolic disorders induced by streptozotocin was demonstrated. These results are consistent with results of our previous study, in which we observed that caffeine at the same dose did not unfavorably affect the skeletal system of female rats with normal estrogen levels [25]. Moreover, in ovariectomized rats, caffeine slightly counteracted the development of skeletal disorders induced by estrogen deficiency [25]. Also, after administration of caffeine in drinking water to healthy mature male rats, no effects on bone histomorphometric parameters in the tibial metaphysis were demonstrated [35,55].

Results of our studies are contradictory to the majority of reports published so far on effects of caffeine on the skeletal system in rats. In vivo experimental studies demonstrated the unfavorable effects of caffeine on prenatal development [27,28,38], and the skeletal system of young, rapidly growing rats [30,32,33,36,41,42], pregnant rats [37], and on bone healing [35,39,40]. A tendency to worsen bone was also observed in old ovariectomized rats [31]. In most of those studies [30,32,33,34,35,36,37,38,39,40,41,42] caffeine was administered in higher doses than that used in the present study; in the rest of the cited studies [27,28,29,31], unfavorable effects of caffeine administered with the diet at doses of 10 or 20 mg/kg body weight were demonstrated. In our study, caffeine was administered once daily. Moreover, this study was performed on young mature rats, unlike the majority of previous studies, performed on developing organisms or in the models of bone healing.

It should be pointed out that long-term administration of coffee in the diet did not unfavorably affect the rat skeletal system [56]. Our results are consistent with that observation, as well as with the recent report on the lack of negative effect of yerba mate drinking (daily caffeine dose of 20–70 mg/kg) on the rat skeletal system [57].

Although the data on the effects of coffee and caffeine on the human skeletal system are inconsistent, coffee drinking (caffeine intake) has been considered an osteoporosis risk factor for many years [58,59,60]. The meta-analysis on the coffee consumption and risk of fractures, published by Liu et al. in 2012 [61], demonstrated an increase in the risk of fractures, especially for women; the data, however, were insufficient to reach a convincing conclusion. The next meta-analysis, published by Lee et al. in 2014 [62], suggested that daily consumption of coffee is associated with increased fracture risk in women and decreased fracture risk in men. However, studies performed on large cohorts of women and men in Sweden, which belongs to countries with highest coffee intake per capita, revealed that high coffee intake was not associated with an increased risk of fractures [18,19]. Moreover, several reports on the beneficial effects of coffee drinking on the skeletal system have been recently published [20,21,22,23]. Consistently, it was demonstrated that light coffee consumption exerted some protective effect against sarcopenia in men [63].

Caffeine has complex mechanisms of action. Caffeine effects are mainly mediated by blockade of adenosine receptor subtypes, but caffeine also targets phosphodiesterases, calcium release ryanodine-sensitive channels in the sarcoplasmic and endoplasmic reticulum, and γ-aminobutyric acid A receptors [64,65]. Caffeine was also reported to exert antioxidant activity [66]. It is well established that caffeine exerts biphasic effects (behavioral stimulation and weak reward at lower doses and anxiety, irritability, discomfort at higher doses) [67]. It was demonstrated that the low dose effects were induced by blockade of adenosine receptors, and the high dose effects were not due to adenosine receptor antagonism [67]. Also, caffeine was reported to exert biphasic dose–response effects on osteogenesis in primary rat adipose-derived stem cells and a mouse bone marrow stromal cell line: pro-osteogenic effects at lower concentrations, and anti-osteogenic effects at high concentrations [68]. Adenosine is known to take part in regulation of bone metabolism [69,70,71,72]; blockade of A1 receptors prevented, for example, bone loss in estrogen-deficient mice [72]. The differential mechanisms of action of caffeine, depending on the dose used, may contribute to the inconsistency in the results of the experimental studies on the skeletal system.

Our results support the notion that moderate caffeine intake does not negatively affect the skeletal system of mature rats. In this and in our previous study [25], we administered caffeine once daily in the dose of 20 mg/kg by oral gavage. Such a dose in the diet was estimated to be equivalent to human consumption of approximately two cups of coffee daily [31]. Although both our studies did not demonstrate unfavorable effects of moderate dose caffeine, it is possible that caffeine, coffee and other products containing caffeine may exert deleterious effects on the skeletal system when used in high doses. Moreover, other coffee constituents, like trigonelline or phenolic acids, may contribute to its skeletal effects [43,44,45]. Results of our experiments indicate that epidemiological or clinical studies on the effects of caffeine/coffee on the skeletal system in patients with diabetes might provide interesting data.

## 5. Conclusions

Results of the present study indicate that caffeine at a moderate dose did not exert damaging effect on the skeletal system of young mature female rats with streptozotocin-induced metabolic disorders.

## Figures and Tables

**Table 1:** Effect of caffeine administered orally (20 mg/kg daily for four weeks) on the serum bone turnover markers and other biochemical parameters in rats treated with streptozotocin (STZ) or nicotinamide and streptozotocin (NA/STZ).

Parameter/Group	Control	STZ	STZ + Caffeine	NA/STZ	NA/STZ + Caffeine
Osteocalcin (ng/mL)	243.6 ± 11.3	149.9 ± 7.9 *	167.4 ± 50.9	287.4 ± 38.4	320.1 ± 26.3 *
C-terminal type I collagen fragments (RatLaps) (ng/mL)	11.29 ± 0.66	49.56 ± 11.32 **	45.29 ± 5.57 *	13.22 ± 1.01	14.33 ± 1.35
Total calcium (mg/100 mL)	9.88 ± 0.09	8.95 ± 0.40	9.80 ± 0.20	10.03 ± 0.17	9.94 ± 0.24
Fructosamine (mmol/L)	0.549 ± 0.012	1.232 ± 0.117 ***	1.184 ± 0.075 ***	0.563 ± 0.020	0.528 ± 0.021
Total cholesterol (mg/100 mL)	36.32 ± 2.28	40.80 ± 3.61	41.95 ± 1.48	34.05 ± 2.43	34.65 ± 3.54

Results are presented as means ± SEM (standard error of the mean; *n* = 8–10). Kruskal–Wallis ANOVA followed by Mann–Whitney U test were used for evaluation of the significance of the results. * *p* < 0.05, ** *p* < 0.01, *** *p* < 0.001—in comparison with the control group.

**Table 2:** Effect of caffeine administered orally (20 mg/kg daily for four weeks) on bone mineral content (BMC), bone area and bone mineral density (BMD) of the tibia (without the proximal epiphysis) and L-4 vertebra in rats treated with streptozotocin (STZ) or nicotinamide and streptozotocin (NA/STZ).

Parameter/Group		Control	STZ	STZ + Caffeine	NA/STZ	NA/STZ + Caffeine
BMC (g)	tibia	0.261 ± 0.005	0.212 ± 0.007 ***	0.224 ± 0.006 ***	0.249 ± 0.005	0.262 ± 0.008
	L-4 vertebra	0.092 ± 0.002	0.069 ± 0.003 ***	0.071 ± 0.003 ***	0.087 ± 0.003	0.094 ± 0.004
Bone area (cm^2^)	tibia	2.163 ± 0.023	2.068 ± 0.029	2.084 ± 0.025	2.139 ± 0.019	2.132 ± 0.025
	L-4 vertebra	0.668 ± 0.014	0.644 ± 0.016	0.622 ± 0.019	0.672 ± 0.012	0.690 ± 0.022
BMD (g/cm^2^)	tibia	0.122 ± 0.002	0.103 ± 0.003 ***	0.107 ± 0.003 ***	0.117 ± 0.002	0.123 ± 0.003
	L-4 vertebra	0.137 ± 0.002	0.108 ± 0.003 ***	0.115 ± 0.004 ***	0.128 ± 0.003 *	0.135 ± 0.003

Results are presented as means ± SEM (standard error of the mean; *n* = 8–10). Kruskal–Wallis ANOVA followed by Mann–Whitney U test were used for evaluation of the significance of the results. * *p* < 0.05, *** *p* < 0.001—in comparison with the control group.

**Table 3:** Effect of caffeine administered orally (20 mg/kg daily for four weeks) on bone mechanical properties in rats treated with streptozotocin (STZ) or nicotinamide and streptozotocin (NA/STZ).

Parameter/Group		Control	STZ	STZ + Caffeine	NA/STZ	NA/STZ + Caffeine
Tibia	Young’s modulus (MPa)	3299 ± 287	2971 ± 340	2340 ± 306 *	3366 ± 252	3704 ± 241
	Maximum load (N)	125.2 ± 7.8	54.5 ± 4.6 ***	50.8 ± 3.9 ***	103.4 ± 4.8 *	109.5 ± 6.5
	Displacement for maximum load (mm)	0.780 ± 0.030	0.604 ± 0.042	0.677 ± 0.062	0.726 ± 0.035	0.819 ± 0.077
	Energy for maximum load (mJ)	48.18 ± 4.65	18.34 ± 2.63 ***	21.21 ± 2.34 ***	39.02 ± 3.44	46.10 ± 6.17
	Stress for maximum load (MPa)	112.4 ± 8.5	57.4 ± 5.9 ***	48.5 ± 4.1 ***	96.3 ± 4.6	105.4 ± 8.6
Femur	Diaphysis—maximum load (N)	121.3 ± 3.8	114.4 ± 3.0	117.5 ± 3.0	118.6 ± 3.9	122.2 ± 4.5
	Neck—maximum load (N)	80.7 ± 1.9	81.7 ± 2.6	79.8 ± 3.5	77.8 ± 3.1	82.6 ± 3.9

Results are presented as means ± SEM (standard error of the mean; *n* = 8–10). Kruskal–Wallis ANOVA followed by Mann–Whitney U test were used for evaluation of the significance of the results. * *p* < 0.05, *** *p* < 0.001—in comparison with the control group.

**Table 4:** Effect of caffeine administered orally (20 mg/kg daily for four weeks) on bone histomorphometric parameters in rats treated with streptozotocin (STZ) or nicotinamide and streptozotocin (NA/STZ).

Parameter/Group		Control	STZ	STZ + Caffeine	NA/STZ	NA/STZ + Caffeine
Tibia	Transverse cross-sectional area of the cortical bone (mm^2^)	3.403 ± 0.074	3.138 ± 0.105	3.235 ± 0.115	3.413 ± 0.072	3.379 ± 0.083
	Transverse cross-sectional area of the marrow cavity (mm^2^)	0.654 ± 0.026	0.757 ± 0.045	0.851 ± 0.072	0.714 ± 0.027	0.707 ± 0.034
	Transverse cross-section of the marrow cavity/diaphysis area ratio	0.162 ± 0.007	0.193 ± 0.004 **	0.207 ± 0.013 **	0.173 ± 0.006	0.173 ± 0.008
Femur	Width of epiphyseal trabeculae (μm)	56.95 ± 1.33	52.90 ± 1.44	53.24 ± 0.80	53.27 ± 1.29	55.72 ± 0.97
	Width of metaphyseal trabeculae (μm)	34.92 ± 1.00	31.29 ± 0.86	32.58 ± 0.93	32.60 ± 1.79	32.90 ± 0.55
	With of epiphyseal cartilage (μm)	46.94 ± 2.82	31.10 ± 2.33 **	34.79 ± 1.94 **	55.64 ± 14.49	51.76 ± 3.94

Results are presented as means ± SEM (standard error of the mean; *n* = 8–10). Kruskal–Wallis ANOVA followed by Mann–Whitney U test were used for evaluation of the significance of the results. ** *p* < 0.01—in comparison with the control group.
